# Smith’s New Label-holder

**Published:** 1882-06

**Authors:** 


Smith's New Label-Holder. Published by Geo. W. Smith, Pharmacist: Cincinnati, Ohio. 1882.

This excellent label-holder "contains 5,250 labels; 736 remedies repeated from five to thirty times, according to the frequency of their use." The labels are ready cut and gummed for use; are pasted on heavy paper so that the leaves do not twist and double up as the labels are used. The type is black and distinct.

